# Multifunctional Applications of Ionic Liquids in Polymer Materials: A Brief Review

**DOI:** 10.3390/molecules28093836

**Published:** 2023-04-30

**Authors:** Liping Wei, Lin Wang, Ziwen Cui, Yingjun Liu, Aihua Du

**Affiliations:** Key Laboratory of Rubber-Plastics (Ministry of Education), School of Polymer Science and Engineering, Qingdao University of Science and Technology, Qingdao 266042, China

**Keywords:** ionic liquids, nanofillers, polymer materials, modification mechanism, multifunctional applications

## Abstract

As a new generation of green media and functional materials, ionic liquids (ILs) have been extensively investigated in scientific and industrial communities, which have found numerous ap-plications in polymeric materials. On the one hand, much of the research has determined that ILs can be applied to modify polymers which use nanofillers such as carbon black, silica, graphene oxide, multi-walled carbon nanotubes, etc., toward the fabrication of high-performance polymer composites. On the other hand, ILs were extensively reported to be utilized to fabricate polymeric materials with improved thermal stability, thermal and electrical conductivity, etc. Despite substantial progress in these areas, summary and discussion of state-of-the-art functionalities and underlying mechanisms of ILs are still inadequate. In this review, a comprehensive introduction of various fillers modified by ILs precedes a systematic summary of the multifunctional applications of ILs in polymeric materials, emphasizing the effect on vulcanization, thermal stability, electrical and thermal conductivity, selective permeability, electromagnetic shielding, piezoresistive sensitivity and electrochemical activity. Overall, this review in this area is intended to provide a fundamental understanding of ILs within a polymer context based on advantages and disadvantages, to help researchers expand ideas on the promising applications of ILs in polymer fabrication with enormous potential.

## 1. Introduction

After more than two decades of rapid development, ionic liquids (ILs), a new generation of green media and functional materials, have become the frontier of current international scientific and technological research and continue to make breakthroughs in industrial applications. Many applications involving ILs include batteries [1], fuel cells, supercapacitors [2], sensors, chemical demulsification [3], catalysts [4], solvents, performance additives [5], and media for molecular self-assembly [6]. The vigorous development of ILs’ research demonstrates the trend of high cross-fertilization of today’s disciplines and the broad development prospect of the application of ILs.

**Figure 1:**
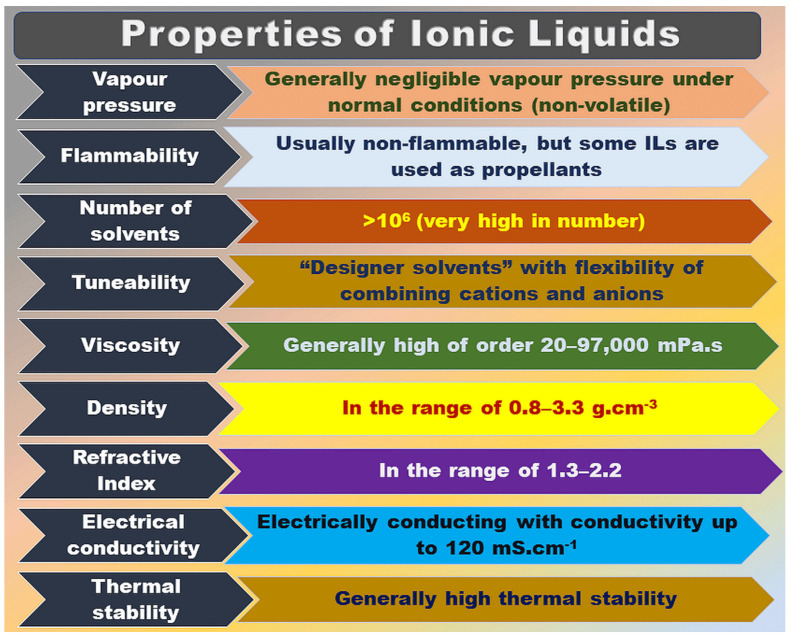
General properties of ILs. Figure adapted from reference [8].

ILs are composed of specific anions and cations, usually consisting of larger organic cations and smaller organic or inorganic anions [7], bound to each other by Coulomb forces. ILs are kinds of salts with a low melting point (<100 °C), and can be utilized as excellent solvents for various polar and non-polar compounds. ILs have many advantages, such as low viscosity, low saturation vapor pressure, high chemical, and thermal stability, non-flammability, wide electrochemical window, and high ionic conductivity, as shown in Figure 1 [8,9]. As for the designer solvents displayed in Figure 1, we can understand that various organic cations with different substituent groups can be combined with anions of interest to design ILs based on the requirements.

With the variety of anions and cations in ILs, there are, theoretically, infinite possibilities for different combinations of the two to obtain an extraordinary variety of ILs. Many researchers have been scientifically and systematically devoted to predicting the structure and properties of ILs and their relationship. Zhang et al. collected relevant data on the physical properties of pure ILs and their mixtures since 1984, and established a database of ILs [10], which consisted of 9400 datapoints and contained 1886 ILs, with 185 anions and 807 cations. Based on the analysis and generalization, they proposed the periodic variation law and orientation diagram of ILs through further study [11], which provided a reference and theoretical basis to select suitable ILs precisely and efficiently.

Due to the high efficiency, excellent performance, and low risk, ILs could replace specific components in many applications; for example, small amounts of ILs could catalyze silanization reactions efficiently and enhance the interfacial interaction of composites. Meanwhile, they are relatively easy to be recycled by extraction and distillation, which reduces their cost in industrial applications and expands their fields of application, such as sensors [12,13,14], batteries [15,16,17,18], ionized gels [19], separation membrane [20,21,22,23,24], etc. Currently, the applications of ILs are becoming more and more extensive in polymer materials, which involve rubber [25,26,27], plastics [28], polyurethane [29,30], epoxy resins [31,32,33], thermoplastic elastomers [34,35], and bio-based polymers [36]. We have summarized the ILs used in polymeric materials in Table 1.

As we can see from Table 1, the commonly-used types of ILs are imidazoles and pyridines in polymer materials. The imidazolium ILs contain alkylimidazolium halide, alkylimidazolium hexafluorophosphate, alkylimidazolium tetrafluoroborate, alkylimidazolium bis(trifluoromethylsulfonyl)imide, alkylimidazolium tetrachloroaluminate, alkylimidazolium acetate, alkylimidazolium dicyanamide, alkylimidazolium mercaptopropinate, and alkylimidazolium mercaptosuccinate. As for pyridines, ILs contain alkylpridinium halide and alkylpridinium tetrafluoroborate. They play many roles in polymer composites, including solvents, vulcanization accelerators, curing agents, dispersants, plasticizers [106], grafting agents, bulking agents, interfacial modifiers, surfactants, and catalysts. In rubber composites, ILs can function as multifunctional modifiers to modify various fillers such as carbon black, silica, carbon nanotubes, multi-walled carbon nanotubes, graphene, graphene oxide, montmorillonite, hydrotalcite, aramid pulp, halloysite nanotubes, powdered cellulose, etc. The dispersion of the fillers is improved in the matrix through the interaction between ILs and the fillers, leading to the improved performance of rubber composites. Next, we will focus on the application of ILs in polymer materials and summarize the mechanism of action of ILs, to provide the theoretical basis for practical applications.

## 2. Filler Modification by ILs

In the rubber industry, rubbers are usually incorporated with many fillers, as most neat rubbers suffer from poor mechanical performance. Fillers confer rubber many excellent properties. For example, reinforcing fillers can not only improve the processing performance of rubber, but also impart the rubber with high strength, improved wear and heat resistance, and other excellent properties, which can extend the service life of products; as for non-reinforcing fillers, they mainly play the role of filling capacity, including isolation, demolding, or coloring. However, inorganic nanopowders are not easily dispersed within the polymeric material matrix. Thus, it is a significant research topic to find economical and straightforward methods to improve their dispersibility and further enhance the interfacial interaction between inorganic nanopowders and matrix materials. This subsection highlights the effect of ILs on the dispersibility of inorganic fillers and interfacial interaction.

### 2.1. Carbon Black

Carbon black is one of the most commonly-used reinforcing fillers in the rubber industry. It is composed of microcrystals with off-domain π-electrons. Aggregates are the most minor structural units of carbon black in rubber matrix, which are usually formed by van der Waals forces. Carbon black can be modified to prevent the formation of agglomerates and improve the interfacial interactions between carbon black and rubber, such as surface adsorption, surface grafting, surface oxidation, etc. Although specific modification effects have been achieved, the modification processes either are expensive or require high solvent consumption, and may destroy the carbon black structure [107].

ILs, as a new kind of green modifier, have been reported to improve the dispersion of nanoparticles within a polymeric matrix [44,92,108] and enhance the interfacial interaction of carbon black and the matrix material, simultaneously. Alkyl imidazole salts containing NTf_2_ anions significantly improved the dispersion of the curing agent and carbon black particles in styrene butadiene rubber (SBR), because ILs reduced the interactions among the carbon black particles, thus reducing their tendency to agglomerate within the polymer matrix [109]. Meanwhile, the interaction of free radicals and off-domain π-electrons on the surface of carbon black particles with cations in ILs can improve the dispersion of carbon black in the matrix and enhance the interfacial interactions between carbon black and the matrix material. The [AMIM] [Cl] was selected to modify the surface of different grades of carbon black. The interaction between the cations of [AMIM] [Cl] and the surface groups of carbon black led to the formation of carbon black–ionic liquid bucky gel, resulting in significant improvement in the mechanical properties of the vulcanization rubber filled with carbon black [44].

**Figure 2:**
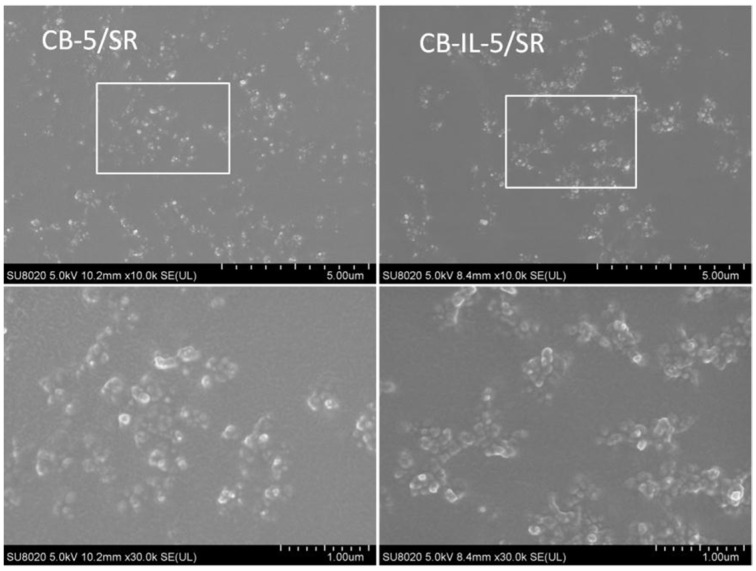
SEM images of the fracture surface of CB-5/SR and CB-IL-5/SR composites (CB-5/SR represents CB-5/SR composite with 5 vol% content of IL, CB-IL-5/SR represents CB-IL-5/SR composite with 5 vol% content of CB-IL). Adapted from reference [80].

A new functionalized IL, composed of mercaptobenzothiazole (MBT) anion and 1-allyl-3-methylimidazolium cation, was used to modify carbon black. Concerning the strong cation–π interaction between imidazole-like ILs and carbon nanotubes and the similar microstructure of carbon black and carbon nanotubes, we infer that the newly-synthesized ILs have a strong interaction with carbon black, improving the dispersion of carbon black within the rubber matrix. As such, the tensile and tear strength of vulcanized filled styrene butadiene rubber (ESBR) composites are significantly improved [107]. After the modification of carbon black with ILs, it was found that the size of the carbon black aggregates became smaller (as shown in the SEM images of Figure 2). Meanwhile, the ILs improved the compatibility between carbon black and silicone rubber (SR), resulting in composites with a lower percolation threshold compared to those of unmodified carbon black [71,80].

### 2.2. Silica

Due to the plentiful number of silanols on the silica surface, the poor dispersion of polar silica in non-polar rubber matrix and weak rubber–silica interfacial interaction are concurrent obstacles for silica utilization in the rubber industry. Toward this dilemma, many efforts have been made, including silane coupling agents [110], physical coating [111], ionizing radiation [112], chemical reactions [113], and macromolecular functionalization [114] methods. With the development of ionic liquid-modified nanofiller technology, many studies have reported that ILs could improve silica dispersion within matrix materials and enhance the interfacial bond strength [39,40,95,115].

**Figure 3:**
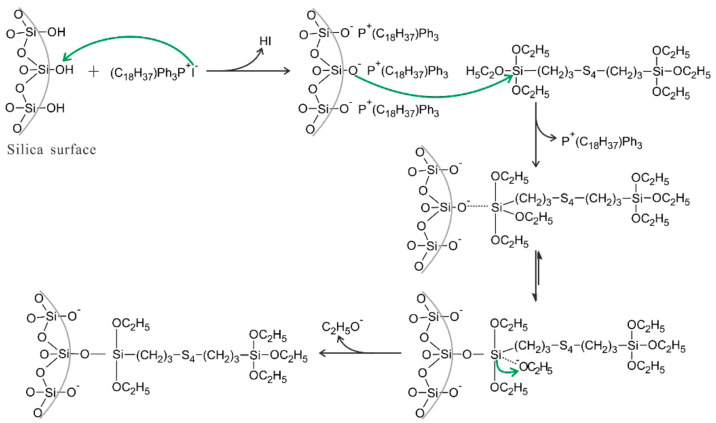
Proposed mechanism for the catalysis of silanization reaction by PIL. The green arrows were the attack route of atoms, and the black arrows were the direction of reaction. Image published in reference [116].

In silica-filled composites, silanization modification has a positive effect on improving the compatibility of silica with the rubber matrix, improving composites’ performance. ILs can be used as catalysts for silanization reactions, which improve the dispersion of silica within the matrix and the interfacial interaction between silica and the matrix material. Phosphorus ionic liquids (PIL), containing trihexyltetradecylphosphonium decanoate and octadecytriphencylphosphonium iodide, were reportedly used as a novel catalyst to promote silanization reactions [116,117]. The addition of a small amount of octadecytriphencylphosphonium iodide can efficiently catalyze the silanization reaction between silica and TESPT by the following mechanism (as shown in Figure 3): The I- anions of PIL can function as a Lewis base catalyst, which react with silica to produce silanolate anions on the surface of silica. The silanolate anions have better nucleophilicity, which facilitates the substitution of the incoming TESPT and promotes the condensation reaction of TESPT with ethoxy to improve the silanization reactions, which improves silica dispersion and dramatically enhances the interfacial interaction between silica and SBR substrate [116].

**Figure 4:**
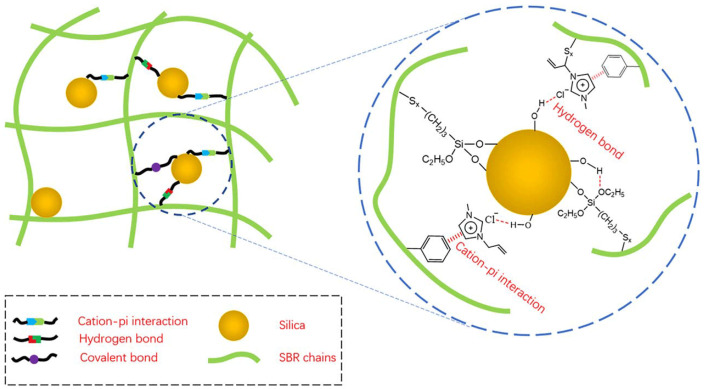
The interaction between silica and SBR chains. Image published in reference [39].

Hydrogen bonding exists between the ILs and the silica surface silicone hydroxyl group, which effectively limits the agglomeration of silica within the rubber matrix [108]. The [AMIM] [Cl] was applied to silica-filled NR and SBR rubber. The infrared spectroscopy, solid state nuclear magnetic resonance, and Raman spectroscopy together indicated that the interaction between [AMIM] [Cl] and silica consisted mainly of hydrogen bonding between Cl^−^ and hydroxyl groups on the surface of silica. Meanwhile, the C=C double bond in [AMIM] [Cl] could be linked to the double bonds of rubber molecules by sulfur bridges to enhance the interaction between silica and rubber (as shown in Figure 4). The interaction parameters of the SiO_2_/NR composites with the addition of ILs were larger than those of the unmodified SiO_2_/NR composites. The larger interaction parameters may be due to the establishment of multiple interactions containing cation–π interactions, hydrogen bonding, and covalent bonding [39,40].

The type of ILs, including cation (imidazolium, pyrrolidinium, and piperidinium) and anion (bromide and chloride), greatly influence the curing characteristics and performance of the rubber composites. Maciejewska et al. [26] investigated the effect of the type of cation and anion on vulcanization characteristics and thermal properties in the NR rubber matrix. They found that the type of cation affected the activity of ILs in the vulcanization, influencing the crosslink density of the vulcanizates. Specifically, the composites containing imidazolium-based ILs, especially with butyl substituent, had the highest crosslink density. Moreover, the thermal stability was also influenced significantly by the cation type of ILs, as the thermal stability of imidazolium salts was the highest compared to other salts, and the most thermally-stable were NR composites containing imidazolium ILs. In the SBR matrix, the ILs ([BMIM] [Br]) showed a beneficial influence on the optimal vulcanization time of SBR/silica compounds, as well as crosslink density and performance of the vulcanizates of SBR/silica, when compared to the other kinds of ILs [53].

### 2.3. Graphene Oxide

Graphene oxide (GO) is a material with a large specific surface area, high mechanical strength, good electrical conductivity, and a unique size effect, making it a potential application in rubber nanocomposites and an ideal filler material. In recent years, GO has been widely used as a filler material for polymers, such as NR, BR, silicone, fluoroether rubber, PLA, and PI, to enhance their mechanical, thermal, thermoelectric, and rheological properties, etc. One of the main problems of rubber composites is achieving the uniform dispersion of the filler within the rubber matrix and the interfacial interaction between the filler and the rubber matrix [118]. Among the many modification methods, the use of ILs to modify the surface of GO and obtain good dispersion is increasingly favored by researchers because of their unique physicochemical properties and environmental friendliness.

Due to the cation–π interaction between the cation in ILs and the π-electrons of the graphite structure in GO, the interaction between the ILs and GO is strong, which improves the dispersion of GO and enhances the interfacial interaction between the graphene and matrix material [100]. For example, graphene can be modified by 1-butyl-3-methylimidazolium tetrafluoroborate ([BMIM] [BF_4_]), and [BMIM] [BF_4_] is embedded in GO layers and attaches to the GO surface through π–π, cation–π, and van der Waals force interactions. Therefore, the distance between GO layers is increased, which improves GO dispersion and interfacial compatibility within the matrix, resulting in excellent tribological, mechanical, and thermal properties of the composites. Specifically, the friction coefficient and wear rate of the composites were reduced by 38.2% and 25%, respectively, compared to those of pure PI [68]. In addition, it has been reported that 1-butyl-3-methyl imidazolium hexafluorophosphate ([BMIM] [PF_6_]) can be inserted into the GO sheet layer effectively to increase the exfoliation degree of GO. After being incorporated with the [BMIM] [PF_6_]-modified GO, the thermal conductivity and thermal stability of BIIR were both significantly enhanced [73].

**Figure 5:**
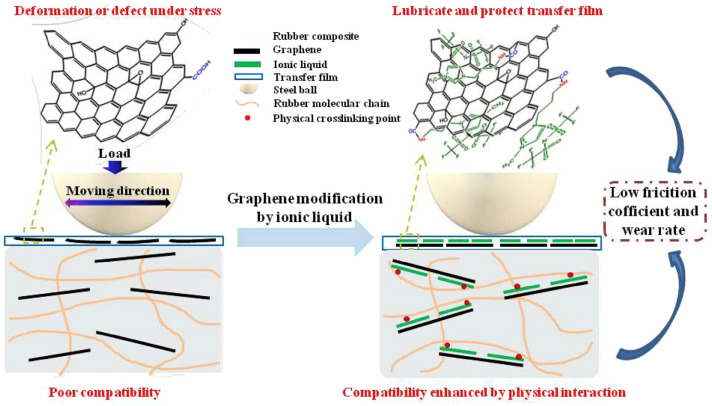
Schematic diagram of tribological properties improvement mechanism of the graphene/fluorother rubber composite induced by IL. Figure adapted from reference [119].

ILs can function as self-lubricating layers of graphene and facilitate the formation of strong interfacial bonds between the ILs-modified graphene and rubber matrix. Based on this function, the QM (silicone rubber) exhibited best wear resistance after being filled with 1.5 phr 1-ethyl-3-methylimidazolium dicyanamide ([EMIM] [Dca])-modified GO [99]. The fluorocarbon- and amino-containing IL 1-(3-aminopropyl)-3-methylimidazolium bis-(trifluoromethylsulphonyl)-imide has also been reported to modify GO used in the context of fluoroether rubber [119]. As shown in Figure 5, the ILs provided an effective self-lubricating layer for the graphene oxide and facilitated the formation of strong interfacial interaction between graphene oxide and the fluoroether rubber, which led to a 13.1% and 59.8% decrease in the friction coefficient and wear rate, respectively, when compared with those of unmodified GO-filled fluoroether rubber [119]. The 1-butyl-1-methylpyrrolidinium hexafluorophosphate ([Bmpyr] [PF_6_]) ILs can also be used as an internal lubricant, increasing the athletic ability of PLA chains in the glassy and rubber states, so the processing properties of PLA were greatly improved, but this effect did not prevail when the composite was melted [120].

### 2.4. Multi-Walled Carbon Nanotubes

Multi-walled carbon nanotubes (MWCNTs) are widely used in various polymer materials due to their good electrical conductivity, thermal conductivity, and mechanical properties. However, because of their high aspect ratio, large specific surface area, and easy agglomeration, their application often fails to achieve the expected results in polymer materials. Thus, the modification of multi-walled carbon nanotubes is a very worthy topic of research from the perspective of dispersibility. To achieve better dispersion of MWCNTs within the rubber matrix, several methods have been successfully obtained by the relevant researchers, including covalent bonding modification and non-covalent bonding modification, in which covalent bonding modification may lead to the destruction of the surface structure of MWCNTs. Many other methods require organic solvents to treat MWCNTs, which can lead to environmental pollution, corrosion of equipment, and health hazards. Therefore, it is necessary to choose environmentally-friendly methods to overcome these drawbacks. ILs, as green solvents, are used to disperse or modify MWCNTs [78,92,121,122,123,124], because the existence of cation–π as well as π–π interactions between them [47,85,125] could effectively reduce the π–π stacking between the particles of MWCNTs.

**Figure 6:**
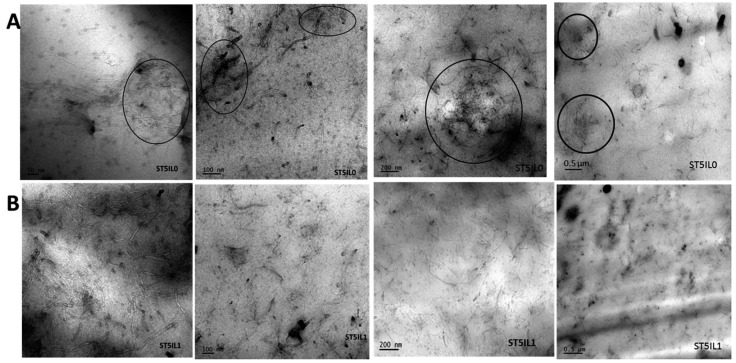
Transmission electron microscopy image of unmodified (**A**) and modified (**B**) composites at different magnifications. The large MWCNTs agglomerates were circled by black circles. Images adapted from reference [125].

Due to the physical interaction (cation–π and π–π interaction) between MWCNTs and ILs, the dispersion of MWCNTs was significantly improved within the NBR matrix, leading to a more uniformly crosslinked rubber network structure, resulting in improved mechanical properties and fatigue resistance. Specially, the composites filled with 3 phr ILs-modified MWCNTs showed the most significant improvement in mechanical properties [57]. Similarly, in the SBR matrix, the dispersion of ILs-modified MWCNTs was better than that of unmodified MWCNTs, as depicted in Figure 6, in which large agglomerates surrounded by black circles were observed in the cross-section of unmodified MWCNTs-filled SBR. Meanwhile, the interfacial compatibility between MWCNTs and SBR was enhanced, resulting in significantly improved tensile strength, hardness, wear resistance, and electrical conductivity of rubber composites [125].

It has been demonstrated that the effect of modifying MWCNTs by two types of ILs simultaneously is better than that of by only one type of IL. MWCNTs modified with a single type of IL formed agglomerates in the ethylene–vinyl acetate rubber (EVM) matrix, while MWCNTs were uniformly dispersed in the EVM matrix when two types of ILs were used to modify MWCNTs simultaneously. The mechanism of this synergistic effect was that EVM was miscible with [TBMA] [NTf_2_] but insoluble with [EMIM] [BF_4_]. Moreover, the interaction of MWCNTs with [EMIM] [BF_4_] was higher than that of [TBMA] [NTf_2_]. The interaction between the ILs, MWCNTs, and EVM was different, so the uniform dispersion of MWCNTs within the EVM matrix was attributed to the bridging effect of the two kinds of ILs, which opened up new avenues for preparing high-performance polymer nanocomposites [70].

### 2.5. Other Fillers

With the development of ecology and the increasing environmental concerns, diverse fillers have been developed to be used in polymer materials, such as starch and walnut shells [50], hydrotalcite [54,82], powdered cellulose [54], microcrystalline cellulose [107], halloysite nanotubes [75,101], aramid pulp [126,127,128,129], etc. After being modified by the ILs, the emerging fillers can impart the polymer composites with new functionalities compared to those of traditional fillers.

Much of the research has demonstrated that the bio-based fillers, such as starch and walnut shell powder modified by ILs ([BMIM] [Cl]), can be used in rubber composites. The dispersion of starch and walnut shell powder was improved significantly [50]. The reason was that ILs could interact with polysaccharides through hydrogen bonding, reducing the interaction of filler particles. Moreover, the addition of [BMIM] [Cl] increased the crosslinking density of the rubber composites. The NR vulcanizate filled by ILs-modified hydrotalcite (HTA) had stronger tensile strength than that filled by other fillers, due to the strengthening effect of HTA and its high crosslinking density [54].

The halloysite nanotubes (HNTs) are a kind of natural clay that feature nano-structure, good thermal stability, and flame retardancy. The hydroxyl functional groups of HNTs are mainly located on the inner surface and tube ends. The distribution of functional groups contributes to the fine dispersion of HNTs in polymer composites. However, the interface interaction between HNTs and polymers is poor and needs to be improved by modification of HNTs. The [MIM] [MP] and [BMIM] [MS], belonging to mercaptan ILs, were used as new interface modifiers. Such mercaptan ILs could be grafted onto the SBR chain through a thiol-ene reaction, promoting the dispersion of HNTs within the rubber matrix effectively [75,101].

Aramid pulp (AP) is widely-used in the rubber industry to provide dimensional stability before vulcanization and improve the mechanical properties of rubber products. ILs, as a kind of compatibilizer, are capable of improving the dispersion of aramid pulp within the rubber matrix. The strong interaction between ILs and AP destroyed the hydrogen bond network between aramid pulp chains, increasing its surface roughness and the carboxyl functionalization. Thus, the interfacial interaction between aramid pulp and the matrix material became strong, making the vulcanizate of the composites have a high modulus under 100% strain with a high breaking strain [130]. At the same time, it was found that the biochar had a synergistic effect with the aramid pulp treated with ILs. The tensile strength of the mixed system was much higher than that of biochar and carbon black. It is predicted that the aramid pulp has potential to partially or entirely replace the carbon black in the formula [126].

## 3. Application of ILs in Polymer Materials

### 3.1. Multiple Functions of ILs in Rubber Vulcanization

A complete sulfur vulcanization system consists of three parts: vulcanizing agent, active agent, and accelerator. Zinc oxide (ZnO) is used as an activator for elastomer vulcanization, with the function of shortening vulcanization time by way of forming zinc complexes with accelerators [131]. At the same time, the rubber composites filled with ZnO could improve the thermal conductivity and prevent the corrosion of rubber by mold organisms and ultraviolet rays, simultaneously. For the protection of biological safety, EU environmental legislation requires that the use of ZnO and zinc-containing compounds be reduced technically. During the rubber vulcanization process, accelerator, sulfur, and stearic acid particles diffuse through the elastomer matrix and attach to the ZnO surface, forming intermediate reaction complexes. Therefore, to improve the vulcanization efficiency, the contact between ZnO and the accelerator in the elastomer matrix should be maximized, which is dependent on the dispersion of ZnO particles within the elastomer. In recent years, ILs have been widely used to improve the dispersion of nanoparticles in polymers due to their unique chemical structure.

**Figure 7:**
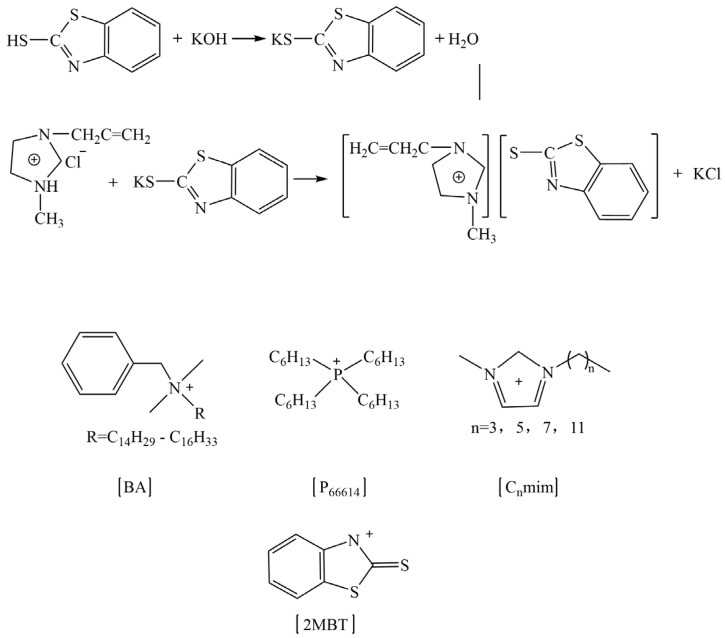
The scheme of prepared ILs based on 2-mercaptobenzothiazolate anion. Image created using data published in reference [132].

Regarding the role of ILs in the rubber vulcanization process, Magdalene et al. have carried out much research and found that ILs could promote rubber vulcanization under the traditional sulfur vulcanization system. As for the reasons, there are three main aspects, as follow: (1) ILs improve the dispersion of active zinc oxide [109,132,133,134]; (2) the ILs act as vulcanization accelerators [132,135]; (3) the ILs act as the active agent, which is similar to stearic acid [109,131]. They synthesized imidazole, benzethonium, and phosphorus ILs, which were based on 1, 3-dialkylimidazolium, benzalkonium (where R = C_12_H_25_ 60% and C_14_H_29_ 40%), and phosphonium cations combined with the 2-mercaptobenzothiazolate anion (the scheme shown in Figure 7), which were used as vulcanization accelerators for nitrile butadiene rubber (NBR), to compare with the use of the conventional vulcanization accelerator M (2-mercaptobenzothiazole). Compared with conventional accelerator M, the use of synthetic ILs as accelerators reduced the process positive vulcanization time of NBR by about half, which significantly improved vulcanization efficiency. At the same time, the particle size of ZnO decreased from around 10 μm to 1–2 μm, and the dispersion of ZnO particles became more uniform in the NBR matrix, which indicated that the synthesized ILs also played a role in promoting the dispersion of ZnO [132]. Meanwhile, the utilization of ILs reduced the amount of 2-mercaptobenzothiazole in the vulcanized rubber, which made the product contain fewer allergens and be friendlier to humans. Specialized ILs have been designed by researchers. The ILs which contained carboxyl groups reacted with ZnO, improving the solubility and dispersion of ZnO particles in the ethylene–propylene-diene monomer (EPDM) matrix, which affected the vulcanization characteristics of the rubber compound [136]. Furthermore, Martyna et al. investigated the role of ILs in the curing reaction of cis-butadiene rubber under a peroxide curing system. The results showed that, via incorporation of ILs, the dispersion of silica was significantly improved. At the same time, the vulcanization time was shortened, as the activation energy of vulcanization was decreased by ILs. Furthermore, ILs catalyzed the interfacial crosslinking reaction to increase the crosslink density [137].

### 3.2. Applications in the Thermal Stability of Rubber

The thermal stability of the nanocomposite depends on the dispersion of the filler within the rubber matrix. The higher the dispersion of the filler, the better the thermal resistance of the nanocomposite [90]. Graphene oxide (GO), which has a good gas barrier property, can effectively prevent the entry of oxygen during the thermal degradation of rubber. At the same time, its two-dimensional lamellar structure can play a role in delaying the thermal degradation of rubber. As such, GO-filled rubber can effectively improve the thermal stability of rubber. However, GO is hard to disperse uniformly in non-polar rubber, resulting in deteriorated rubber performance. The thermal stability of rubber composites can be improved by enhanced GO dispersion by modification via ILs, because the ILs effectively embed into the interlayer of GO to improve its peeling degree [73].

The literature has reported that, in carbon black-filled EPDM composites, the structure of the ILs, including the alkyl length bonded to the imidazolium and the type of anion, has a significant effect on EPDM rubber composites, including rubber vulcanization, thermal stability, and dynamic mechanical properties [127]. The ILs containing long alkyl chains elevate the onset vulcanization temperature and lower the vulcanization enthalpies. Additionally, the poor thermal stability of the pure ILs reduces the onset of decomposition temperature of the EPDM composites. Moreover, the onset decomposition temperature of pure ILs increases with the length of alkyl chains in the imidazolium cations. Among all the ILs, the lowest onset decomposition temperature has been observed for ILs containing chloride anions. As for the dynamic mechanical properties, the ILs’ type of anion seems to be crucial. The storage modulus of vulcanizates containing alkylimidazolium chloride was slightly lower or comparable to that of EPDM without ILs in the glassy state. In contrast, vulcanizates containing ILs with BF_4_ and PF_6_ anions had a storage modulus about 100–300 MPa higher than that of reference vulcanizates. Kalaivani et al. investigated the thermal degradation of unmodified and ILs-modified MWCNTs-filled chloroprene rubber (CR) composites under nitrogen and air conditions. The thermal stability showed the following trend: CR < unmodified MWCNTs/CR composites < ILs-modified MWCNTs/CR composites. This trend was attributed to the enhanced interfacial interaction between the ILs-modified MWCNTs and the CR and the improved dispersibility of the modified MWCNTs in the CR [90]. The thermal stability of the vulcanizates is closely correlated with the thermal stability of pure ILs. As well, the type of cation and the length of substituent have an important influence on the thermal stability of pure ILs. Sowinska et al. studied ILs consisting of the same bis (trifluoromethylsulfonyl)imide (NTf_2_) anion, but possess different cations, such as alkylpyrrolidinium, alkylammonium, and alkylsulfonium, with different lengths of alkyl chains. The result showed that those with pyrrolidinium and ammonium cation exhibited much better thermal stability than ILs with sulfonium cation [27].

### 3.3. Applications in the Thermal Conductivity of Rubber

Thermally-conductive rubber can provide an effective heat dissipation path, shock absorption, and insulation, expanding rubber’s application into aerospace, electronic devices, automotive tires, etc. The thermal conductivity of rubber itself is poor, so the research of thermally-conductive rubber formulations mainly lies in selecting the filling system. The thermal conductivity of rubber products depends on the nature of the filling system itself and its distribution within the rubber matrix.

Inorganic materials in polymers have no or very low thermal conductivity; however, they have many internal voids. Therefore, the thermal conductivity of these inorganic fillers can be improved by modification with high thermal conductivity ILs [138]. ILs improved the dispersion of graphene within the matrix material through hydrogen bonding and cation–π interactions, and the thermal conductivity of the composite with 5 phr ILs-modified graphene was improved by 34% compared with the pure matrix material [139]. Compared with that of unmodified MWCNTs-filled HXNBR/HNBR, the thermal conductivity of HXNBR/HNBR composites filled by 7 phr [BMIM] [Cl]-modified MWCNTs was increased by 29% [89].

### 3.4. Applications in Electrical Conductivity of Rubber

High ionic conductivity is one of the most important electrochemical properties of ILs. At room temperature, the conductivity of ILs is generally at the 10^−1^ mS/cm level, making improvement of the electrical conductivity of rubber a huge application potential.

The performance of the ternary polymer electrolytes is related to the ionic conductivity, electrochemical stability, and thermal stability of the ILs used [1]. Vries et al. studied many kinds of ternary polymer electrolytes, consisting of polyethylene oxide (PEO), lithium trifluoromethylsulfonimide (LiNTf_2_), and different ILs. These ILs were a combination of two cations (Pyr_14_ and Pyr_12O1_) and four anions (FSI, NTf_2_, BETI, and IM_14_). For three of the four anions, the total ionic conductivity was increased with Pyr_12O1_, which was compared to the corresponding Pyr_14_ ILs. The smaller the anions, the higher the ionic conductivity (FSI > NTf_2_ > BETI > IM_14_). The ether moiety in the side chain of the pyrrolidinium increased the ionic conductivity, but, in some cases, lowered the thermal and electrochemical stability. Samples with NTf_2_, BETI, and IM_14_ were found to be fully amorphous [140]. Joost et al. found that crosslinked ternary solid polymer electrolytes (SPEs), which consisted of PEO, lithium bis(trifluoromethylsulfonyl)imide (LiNTf_2_), and N-butyl-N-methyl-pyrrolidinium bis(trifluoromethylsulfonyl)imide(Pyr_14_NTf_2_) ILs, had high thermal stability in nitrogen (>300 °C) and oxygen atmospheres (>150 °C), to make the present SPE systems promising candidates for safe batteries [141].

**Figure 8:**
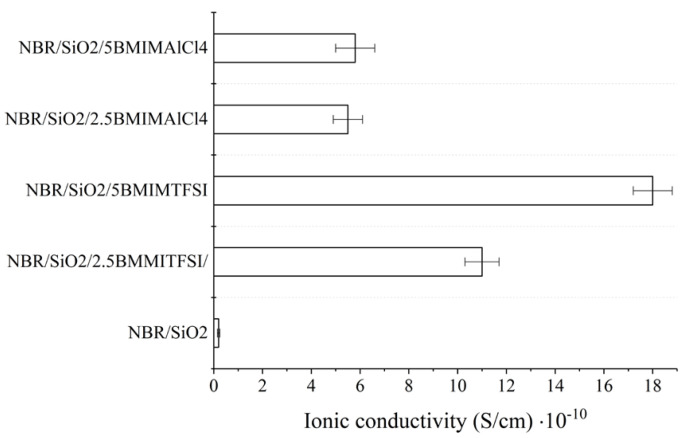
Ionic conductivity of NBR–ionic liquid-based composites measured at room temperature. Image created using data published in reference [81].

Suradet et al. investigated the electrical conductivity of ENR/COPA blends by adding ILs-modified MWCNTs before and after dynamic vulcanization (BDV and ADV). As for BDV and ADV, MWCNTs prioritized locations in the COPA phase. However, some multi-walled carbon nanotubes were also situated in the ENR domain under BDV. Compared with those of TPV prepared by ADV, TPV prepared by BDV showed higher conductivity, dielectric properties, and better stress relaxation behavior, which may be due to the better dispersion of MWCNTs in the two phases of the blend. In addition, with the increase of ILs’ load, electrical and dielectric properties of the elastomer material were both improved [59]. The ionic conductivity of composites usually depends on the electrical conductivity and ionic concentration of pure ILs. When two different imidazole-based ILs ([BMIM] [NTf_2_] and [BMIM] [AlCl_4_]) were added to the NBR/SiO_2_ composites, they increased the ionic conductivity of the composites (as shown in Figure 8). The conductivity of hydrophobic [BMIM] [NTf_2_] was 3.5 mS/cm (25 °C), and that of hydrophilic [BMIM] [AlCl_4_] was 9.2 mS/cm (25 °C). Compared to the NBR/SiO_2_ composite, the ionic conductivity of the NBR/SiO_2_ composites containing 2.5 and 5 phr [BMIM] [AlCl_4_] increased to 5.6 × 10^−12^ to 5.5 × 10^−11^ and 5.8 × 10^−11^ S/cm, respectively. In contrast, the conductivities of the composites containing [BMIM] [NTf_2_] salts were 1.1 × 10^−9^ S/cm (2.5 phr) and 1.8 × 10^−9^ S/cm (5 phr), respectively. This result may be related to the active participation of the AlCl_4_ anion during the crosslinking process and the lower compatibility of ILs with hydrophobic polymers [81].

Qiong et al. obtained graft polymers by reacting the carboxyl group in ILs ([(HOOC)C_1_C_1_Im] [NTf_2_]) with the epoxy group in epoxy natural rubber, and then introduced LiNTf_2_. Next, they successfully prepared solid electrolyte composites with high ionic conductivity, in which the maximum ionic conductivity reached 3.01 × 10^−4^ S/cm (23 °C) in the experimental range [77]. Furthermore, a conductive solid electrolyte was obtained, in which NBR was the matrix, with ILs as plasticizer and triallyl cyanurate as crosslinking agent. The nitrogen atoms in the triallyl trimellitrate contributed to the dissociation of the lithium salt and promoted the dissociation and migration of lithium ions, resulting in an electrolyte with a high ionic conductivity at room temperature [15].

### 3.5. Applications in Polymeric Selectity and Permeability

It is an essential problem in natural and technical processes to separate components from mixtures. For one thing, most substances exist in the form of mixtures in nature, and some of which require separation and purification before being used by humans, such as, water, edible salt, and microorganisms. For another, the separation of mixtures is indispensable in chemical production. Furthermore, combination of membrane processes with ILs has received more and more attention in impurity removal, because they enhance separation efficiency, and also broaden their research and application areas [142]. The realization of a membrane separation process allows for low-energy separation of mixtures. There are three methods for membrane separation: vapor permeation, gas permeation, and pervaporation. As for different membrane separation methods, there are different applications. Polymeric membranes used for gas separation must have specific properties, such as high permeability and selectivity for the desired gas. The unique physicochemical properties of ILs lay a foundation for their application in the direction of polymeric membranes. Relevant research reports indicate that polymeric membranes are formed through mixing of ILs with various polymers in appropriate solvents, and the gas transport performance of membranes can be adjusted by changing the amount of ILs added. Furthermore, ion gels formed by low molecular weight organic gelators and a low concentration of polymer obtain high-efficiency membrane permeability through some methods, for example, using ABA triblock copolymer and soaking an existing membrane in an excess of ILs.

**Figure 9:**
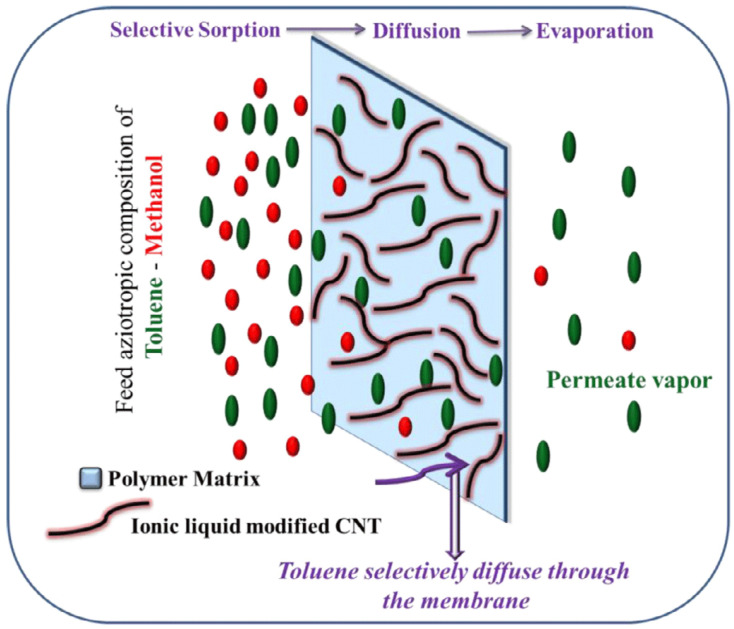
Schematic representation of the mechanism of pervaporation separation of liquid mixtures through MWCNTs’ network structure. The red and green circles were representative of methanol and toluene, respectively. Figure adapted from reference [143].

The vapor permeation (VP) and gas permeation properties of the NR/CNTs/ILs composite membranes were investigated. It was found that the permeability of the membranes of the three systems—NR, NR/CNTs, and NR/CNTs/ILs—to oxygen (O_2_) and nitrogen (N_2_) was highly variable, in which the NR/CNTs/ILs membrane had a high gas permeability of 63.53 and 59.77 Barrers for O_2_ and N_2_, respectively; while in the NR/CNTs membrane, O_2_, and N_2_ permeabilities were 30.5 and 27.72 Barrers, respectively. The reason was the increased free volume of NR/CNTs/ILs membrane, due to the plasticizing properties of ILs. Furthermore, vapor permeation of NR/CNTs/ILs membrane was also successfully applied to the low-cost separation of benzene/cyclohexane azeotropic mixtures [20]. When suitable nano-fillers were added, the polymeric membrane material improved the overall selectivity [22]. Jiji et al. successfully prepared SBR/MWCNTs (CNTs)/ILs membranes to separate toluene from toluene/methanol or toluene/heptane mixtures by permeation vaporization. The mechanism ias shown in Figure 9, where the role of ILs in the composites is to functionalize MWCNTs (CNTs) and be used as plasticizers [63,143].

IL gels enable the transport properties and mechanical properties of the membranes to be tailored over a wide range by variation of the ILs’ content from neat polymer to 80%. The physical crosslinks, which are responsible for the gelation of the polymer/ILs blend, are formed by crystalline domains in the semi-crystalline polymers and by microphase separation and/or crystallization in the thermoplastic elastomers [144]. Nguyen et al. [145] prepared gelled ILs membranes of either [C_6_MIM] [NTf_2_] or [C_2_MIM] [NTf_2_], with different contents (1.5, 3, and 6 wt%) of an aspartame-based low molecular weight organic gelator, and evaluated their mechanical and gas transport properties. These gelled ILs membranes showed a trade-off between good mechanical stability and high gas permeability as a function of the gelator loading, in which an increase in the loading induced a stronger gelator fiber network. Still, it also slowed down gas diffusion in the gel matrix, thus sacrificing gas permeability. Compared to [C_2_MIM] [NTf_2_], the trade-off was observed to be more pronounced for [C_6_MIM] [NTf_2_], probably because the longer carbon chains attached to the imidazolium cation caused less mobility for the ILs’ molecules and stronger interactions with the gelator molecules. Gu et al. [146] examined the gas separation performance of two block copolymer ion gel systems, which were based on 15 wt% poly(styrene-b-ethylene oxide-b-styrene) (SOS) or poly(styrene-b-methyl methacrylate-b-styrene) (SMS) and [C_2_MIM] [NTf_2_]. The supported ion gel membranes exhibited CO_2_ permeabilities between 710 and 840 Barrers, and, due to the high free ILs’ concentration in the system, both SMS and SOS ion gels showed very high CO_2_ permeability. Moreover, the SOS gel exhibited a much higher selectivity for both gas pairs (CO_2_/N_2_, and CO_2_/CH_4_) than both the SMS gel and neat ILs, indicating that the gas separation performance of ion gels could be significantly influenced by the mid-block identity.

As for ion gelation, relevant experts have certified that it is a suitable method to prepare supported pseudo-solid membranes, with good CO_2_ separation performance similar to pure ILs while exhibiting improved mechanical strength. The gelled IL membranes are promising materials for CO_2_ separation that bridge ILs and solid polymers. However, using a matrix reduces their potential CO_2_ separation efficiency, because the CO_2_ permeance of the prepared gelled IL membranes is limited by the thickness of the used matrix [147].

### 3.6. Application of ILs in Electromagnetic Shielding

With the development of electronic information technology and the widespread use of electronic devices, electromagnetic interference (EMI) has become a severe problem, which interrupts the function of electronic devices and seriously affects human organs. Conductive rubber composites filled with carbon materials, such as carbon black and MWCNTs, are widely-used as shielding materials for EMI because of their excellent electrical conductivity and flexibility. Because of the cation–π interactions between the ILs and the carbon fillers, ILs have been used to improve the dispersion of carbon fillers in polymer matrices [34,65,148].

Composites of silicone rubber (SR)/POE blends, filled with carbon black and MWCNTs modified with ILs, were prepared by melt-blending and hot-pressing methods. An SR/POE/CB-CNTs–ILs composite has higher EMI shielding than an SR/POE/CB–ILs composite. This is because MWCNTs have large lengths and specific surface areas, leading to stronger cation–π interactions with ILs than those between carbon black and ILs, which, together with the larger length–diameter ratio of MWCNTs, concurrently contributed to the formation of conductive networks [34]. The effects of the addition of MWCNTs modified by ILs and the phase structure of the composites on the shielding performance were also investigated: (1) With the increase in modified multi-walled carbon nanotubes addition, the EMI shielding performance of the composites significantly improved, because MWCNTs–ILs could form more conductive networks and increase the leakage current; (2) The shielding performance of the composites with co-continuous structure was higher than that of the island structure due to the homogeneous dispersion of MWCNTs–ILs in the co-continuous structure, which improved the electrical conductivity [35].

### 3.7. Application of ILs in Piezoresistive Sensitivity

In the field of sensors, much attention has been given to the application of the piezoresistive behavior of conductive polymer nanocomposites (CPNCs). CPNCs are frontrunners in strain sensor applications; the reason is that they have some advantages, such as facile fabrication, tunable piezoresistive sensitivity, and the capability of sustaining high-level stress/stain. One challenge is to achieve high sensitivity and high ductility/toughness simultaneously in CPNCs-based piezoresistive strain sensors [61]. We could use conductive fillers such as carbon nanotubes, carbon black, graphite, or their nanoscale derivatives to obtain superior mechanical deformation for the composites. Furthermore, the dispersion of filler plays a decisive role in the mixture. The use of ILs allows improvement of the filler dispersion, rubber–filler interaction, and flexibility of the composites, which enhances the piezoresistive performance and sensibility [12].

Kai et al. fabricated the highly piezoresistive and ductile poly (vinylidene fluoride) (PVDF)-based CPNCS, and the interaction between the polymer matrix and MWCNTs was regulated by using ILs (1-butyl-3-methylimidazolium hexafluorophosphate, [BMIM] [PF_6_]) as interface linker/modifier. The addition of ILs achieved uniform dispersion of carbon nanotubes within PVDF, with higher electrical contact resistance, greatly improved piezoresistive sensitivity, and gauge factors ranging from 7 up to 60 [61]. For the silicone rubber filled with carbon black, the dispersion of carbon black in the matrix was improved under the action of ILs (1-hexadecyl-3-methylimidazolium bromide). Compared with CB/SR composite, CB–ILs/SR composite had a lower percolation threshold, higher piezoresistivity, better cyclic repeatability, and shorter relaxation time [80]. At the same time, the ILs could increase response consistency and the flexibility of the composites [45,46]. These features will have great application prospects in flexible stress sensors and wearable electronic devices.

### 3.8. Multiple Functions of ILs in Energy

As a green solvent, ILs possess several advantageous properties, including a wide electrochemical window (ESW), high electrochemical and thermal stability, negligible volatility, intrinsic ionic conductivity, non-flammability, and good solvation capabilities, enabling them to be versatile candidates in various advanced electrochemical applications, such as batteries and fuel cells.

Chen et al. selected [BMIM] [Cl] as the ionic liquid-based aqueous electrolyte due to its high water-soluble and non-electrochemically active nature. The electrolytes were promising for safe high voltage and high energy density applications, owing to the high ESW (3~4.4 V), high ionic conductivity (>10 m S cm^−1^), and excellent flowability (viscosity < 10 m Pa s). Not only did the use of [BMIM] [Cl] in flow batteries provide high stability against decomposition, but also the chloride ions in [BMIM] [Cl] served as charge carriers when used as a separator in anion exchange membranes [51,52]. In order to obtain the operating potential window, Matsuda et al. chose tetrabutylammonium hexafluorophosphate ([TBA] [PF_6_]) as a supporting electrolyte, achieving a maximum cell potential of 2.3 V and a maximum solubility of approximately 0.9 M in redox flow battery (RFBs) [104]. Additionally, Zhang et al. utilized tetraethylammonium hexafluorophosphate ([TEA] [PF_6_]) and 1-ethyl-3-methyl imidazolium hexafluorophosphate ([EMIM] [PF_6_]) as supporting electrolytes, to achieve a potential range of 3.6 V (−2.5~1.5V) [105].

Electrolyte formulation modification is a very important methodology to obtain high performance in RFBs. Specifically, the stability of electrolytes is largely dependent on the electrolyte composition [2]. For [BMIM] [Cl], the reduction potential of the chloride anions and the cations of BMIM is relatively low, and it is considered to be a suitable electrochemical stabilization additive [51,52]. At the same time, the Coulomb repulsion and steric hindrance of BMIM used as surfactant cationic additive could improve the dispersibility of the electrolyte [149]. Li et al. reported that [BMIM] [BF_4_] was used as an additive in the anode electrolyte of vanadium RFB (VRFB). When 1.0% [BMIM] [BF_4_] was added, the electrochemical activity and stability of the anode electrolyte were largely improved, and the energy density and energy efficiency were also increased by incorporating [BMIM] [BF_4_] [150]. In addition, ILs are also used as alternative reaction media to conventional organic solvents for the fabrication of inorganic materials, and play a key role in the synthesis of various cathode and anode active materials [151].

## 4. Summary and Outlook

Because of the diversity of the constituent anions and cations, ILs can be formed by different anion–cation combinations, with infinite possibilities, in principle. In polymer composites, the most commonly-used ILs are imidazoles and pyridines, at present. Nanofillers usually need to be modified to improve their dispersion, as they tend to aggregate within the polymer matrix. For carbon nanofillers, such as carbon black, GO, and MWCNTs, ILs reduce the intermolecular interactions between the nanofiller particles by cation–π interactions (or π–π interactions), because the interaction between cations of ILs and electrons of graphite structures reduces its tendency of filler aggregation in the polymer matrix. Furthermore, ILs can function as a self-lubricating layer of graphene, building a strong interfacial layer of graphene–ionic liquid within the rubber matrix. At the same time, they can effectively insert into the graphene oxide sheet layer to increase the degree of exfoliation of graphene oxide, resulting in enhanced interfacial compatibility with the rubber. Compared with carbon nanofillers, ILs play a much more complex role in silica-filled composites, either as catalysts for silanization reactions or as interface compatibilizers, based on cation–π interactions, hydrogen bonds, and covalent bonds.

In recent years, with the rapid development of ILs’ technology, their application in polymer materials has become more and more extensive, ranging from dispersants, vulcanization accelerators, and activator-like roles in rubber vulcanization to dispersing inorganic fillers in a high thermal and electrical conductivity material, with increasing heat resistance and electrical and thermal conductivity of composite materials. They have been developed to be used as modifier and plasticizer to increase the free volume of polymer membrane material, to improve their overall selectivity increase, which can be successfully used to separate gas and liquid mixtures.

ILs have been utilized as functional additives (such as vulcanization accelerators, curing agents, dispersants, plasticizers, and grafting agents), catalysts, solvents, and electrolytes in various applications, which have been summarized and reviewed in this manuscript. The effective application of ILs requires selecting cations and anions elaborately. For example, the safer and longer service life electrolytes made by conductive rubber materials are desired currently, which can be achieved by regulating the anion structure of ILs. Organic/inorganic hybrid electrolytes incorporated with ILs are also specified for these applications. For the industrial application of ILs, it is always preferable to use ILs with low costs and good recyclability. Therefore, the high price of ILs is the most crucial aspect limiting their application. The production of cheap and easily accessible ILs with multiple functionalities is urgently needed. Furthermore, effective communication between industry and academia will also facilitate the utilization of ILs at the commercial scale.

## Figures and Tables

**Table 1:** Summary of ILs commonly-used in polymeric materials.

ILs	Abbreviation	Application Field	References
1-allyl-3-methylimidazolium chloride	[AMIM] [Cl]	dispersant, solvent	[37,38,39,40,41,42,43,44]
1-decyl-3-methylimidazolium chloride	[DMIM] [Cl]	dispersant, sensor	[12,45,46,47,48]
1-methylimidazole chloride	[MIM] [Cl]	dispersant, modifier	[30,49]
1-butyl-3-methylimidazolium chloride	[BMIM] [Cl]	membrane separation, solvent, plasticizer, surfactant, conductive, battery	[20,50,51,52]
1-butyl-3-methylimidazolium bromide	[BMIM] [Br]	dispersant, solvent, conductive	[53,54,55]
1-ethyl-3-methylimidazolium chloride	[EMIM] [Cl]		[56,57,58,59]
1-ethyl-3-methylimidazolium bromide	[EMIM] [Br]		[60,61]
1-benzyl-3-methylimidazolium chloride	[BZMIM] [Cl]	membrane separation	[62,63]
1-benzyl-3-ethylimidazolium chloride	[BZEIM] [Cl]		[13,64,65]
1-butyl-3-methylimidazolium tetrafluoroborate	[BMIM] [BF_4_]	dispersant, plasticizer, conductive, membrane separation, battery	[22,66,67,68,69,70]
1-butyl-3-methylimidazolium hexafluorophosphate	[BMIM] [PF_6_]		[19,61,66,71,72,73,74,75]
1-ethyl-3-methylimidazolium tetrafluoroborate	[EMIM] [BF_4_]	dispersant, conductive, electrolyte	[16]
1-carboxyethyl-3-methylimidazolium bis(trifluoromethysulfonyl)imide	[CEMIM] [NTf_2_]	dispersant, conductive, grafting agent, battery	[76,77]
1-hexyl-3-methylimidazolium hexafluorophosphate	[HMIM] [PF_6_]	dispersant, plasticizer, conductive, coupling agent	[71,72,78,79]
1-hexadecyl-3- methylimidazolium bromide	[HDMIM] [Br]	dispersant, conductive	[47,80]
tributylmethylammoniu bis(trifluoromethylsulfonyl)imide	[TBMA] [NTf_2_]		[70]
1-butyl-3-methylimidazolium bis(trifluoromethylsulfonyl)imide	[BMIM] [NTf_2_]	conductive, electromagnetic interference, vulcanization	[81,82,83,84,85,86,87,88,89,90,91,92]
1-propyl-3-methylimidazolium bis(trifluoromethylsulfonyl)imide	[PMIM] [NTf_2_]	vulcanization	[15]
1-ethyl-3-methylimidazolium bis(trifluoromethylsulfonyl)imide	[EMIM] [NTf_2_]	dispersant, electromagnetic interference	[93]
1-vinyl-3-ethylimidazolium bromide	[VEIM] [Br]		[34,35,94]
1-butyl-3-methylimidazolium tetrachloroaluminate	[BMIM] [AlCl_4_]	dispersant	[81,82]
1-ethyl-3-methylimidazolium acetate	[EMIM] [Ac]		[95,96,97,98]
1-ethyl-3-methylimidazolium dicyanamide	[EMIM] [Dca]	catalyst	[99,100]
1-methylimidazolium mercaptopropinate	[MIM] [MP]	dispersant, catalyst, modifier, vulcanization	[101]
bis(1-methylimidazolium) mercaptosuccinate	[BMIM] [MS]		[101]
1-butylpridinium bromide	[BBP] [Br]	dispersant, modifier	[102]
4-methyl-1-butylpyridinium bromide	[BMBP] [Br]		[102]
1-butyl-4-methylpyridinium tetrafluoroborate	[BMP] [BF_4_]		[103]
tetrabutylammonium hexafluorophosphate	[TBA] [PF_6_]	electrolyte	[104]
tetraethylammonium hexafluorophosphate	[TEA] [PF_6_]		[105]
1-ethyl-3-methyl imidazolium hexafluorophosphate	[EMIM] [PF_6_]		[105]

## Data Availability

Not Applicable.
